# Drought Stress Limits the Photosynthetic Benefit of Elevated CO_2_ in Chinese Fir Saplings via Stomatal Closure and Non-Stomatal Impairment

**DOI:** 10.3390/plants15152353

**Published:** 2026-07-30

**Authors:** Yujie Wu, Zhiwei Zhang, Wenjuan Guo, Fulin Chen, Yanghui Fang, Shubin Li, Liang Fang, Linfeng Li

**Affiliations:** 1 College of Forestry, Fujian Agriculture and Forestry University, Fuzhou 350002, China; 2 Fujian Provincial Forestry Science and Technology Promotion General Station, Fuzhou 350003, China

**Keywords:** elevated CO_2_, drought, photosynthesis, stomatal limitation, oxidative stress

## Abstract

The frequency and magnitude of droughts are increasing concurrently with atmospheric CO_2_ concentration, with profound consequences for plant carbon assimilation. However, the interactions and the underlying physiological mechanism are still not fully understood. To fill the knowledge gap, we exposed Chinese fir (*Cunninghamia lanceolata*) saplings to two CO_2_ concentrations (400 and 800 ppm, representing ambient and elevated CO_2_) and two soil water regimes (70% and 40% field capacity; well-watered and drought-stressed conditions) in a factorial design. Net photosynthetic rate (*A*_n_), chlorophyll fluorescence, photosynthetic pigments, oxidative stress indicators, and antioxidant enzyme activities were measured four times over a 45-day treatment period. Under well-watered conditions, elevated CO_2_ significantly increased *A*_n_ by 61.5%. However, drought stress substantially reduced *A*_n_ by 75.0% under ambient CO_2_ and by 75.6% under elevated CO_2_, whereas no statistically significant CO_2_-induced increase was detected under drought conditions. Furthermore, drought stress caused marked reductions in stomatal conductance, transpiration, chlorophyll content, and photosystem II (PSII)-related parameters, together with increased malondialdehyde, proline, and antioxidant enzyme activities. Variance partitioning analysis suggested that stomatal regulation (SR), photosynthetic capacity (PC), and stress response (STR) jointly explained 71% of the variation in *A*_n_. Structural equation modeling further suggested that drought stress restricted the photosynthetic benefits of elevated CO_2_ primarily through stomatal closure, concurrently associated with stress-related declines in pigment stability and photochemical performance. These findings suggest that the carbon sink potential of Chinese fir plantations under future CO_2_-enriched climates may be strongly constrained by water deficits.

## 1. Introduction

Atmospheric carbon dioxide (CO_2_) concentrations have risen sharply and are projected to exceed 800 ppm by the end of this century under high-emission scenarios [1,2]. Meanwhile, the frequency and intensity of drought events are increasing, even in traditionally humid subtropical regions [3,4]. Generally, elevated CO_2_ enhances the carboxylation efficiency of ribulose-1,5-bisphosphate carboxylase/oxygenase (Rubisco) and suppresses photorespiration, thereby stimulating plant carbon fixation (i.e., CO_2_ fertilization effect) [5,6]. In contrast, water deficit is now widely recognized as one of the major environmental constraints on plant photosynthesis and long-term ecosystem carbon sequestration [7,8]. Because carbon acquisition is inherently coupled with water loss through stomata, plant responses to elevated CO_2_ and drought are governed by a fundamental carbon–water trade-off [9,10]. Thus, determining the interaction of elevated CO_2_ and drought on photosynthesis has become a key issue for predicting future ecosystem carbon sequestration [11,12].

The interaction between elevated CO_2_ and drought is highly context-dependent. Under mild or moderate drought, elevated CO_2_ often induces partial stomatal closure, reduces stomatal conductance and transpiration, improves intrinsic water-use efficiency, and delays soil water depletion, thereby mitigating drought stress to some extent [13,14]. However, this protective effect may be ineffective under severe water deficit. In addition to stomatal closure, drought stress induces photosynthetic decline through non-stomatal impairments [15]. For example, disruption of cellular homeostasis promotes excessive accumulation of reactive oxygen species (ROS), which can trigger membrane lipid peroxidation, accelerate photosynthetic pigment degradation, and impair photosystem II (PSII) activity [16,17]. In this case, the interactive effect of elevated CO_2_ and drought depends not only on stomatal behavior and carbon substrate availability, but also on the structure and activity of the photosynthetic apparatus, pigment homeostasis, and photochemical performance [18,19]. Although most studies emphasize stomatal regulation, we integrate both stomatal and photochemical responses into a unified framework. Notably, we define “non-stomatal impairments” strictly as photochemical damage and pigment degradation, excluding internal biochemical limitations (e.g., Rubisco activity). Thus, the CO_2_ fertilization benefits may be strongly constrained once water deficit exceeds the photosynthetic tolerance threshold.

Currently, studies distinguishing the mechanisms underlying the combined effects of elevated CO_2_ and drought on photosynthesis approach this mainly from the perspective of stomatal regulation, whereas fewer studies have integrated stomatal behavior, oxidative stress, pigment stability, and photochemical performance into a unified mechanistic framework [15,16,17,18,19]. This knowledge gap is particularly important for drought-sensitive plantation species, because the CO_2_ fertilization effect may not simply weaken under drought stress, but may be strongly constrained once water deficit exceeds the physiological tolerance threshold of the photosynthetic system. In other words, a key unresolved question is whether severe water deficit merely attenuates the positive effect of elevated CO_2_ or completely overrides it through the combined action of stomatal closure and photochemical impairment.

Chinese fir (*Cunninghamia lanceolata*) is one of the most important fast-growing plantation conifers in subtropical China and plays a major role in timber production, ecological restoration, and regional carbon sequestration [20,21,22]. The productivity of this species depends strongly on favorable hydrothermal conditions [23]. Ecologically, Chinese fir has a relatively shallow root system and limited access to deep soil water, which makes it highly sensitive to declines in soil water availability [24,25]. Meanwhile, seasonal drought events have become increasingly frequent in southern China under recent climate change, exposing Chinese fir plantations to substantial water-deficit risk during active growth periods [26]. Prolonged drought can markedly suppress photosynthetic carbon assimilation and biomass accumulation in Chinese fir and may even threaten plantation stability [12,27]. While the detrimental physiological effects of isolated drought on Chinese fir are well documented, its interactive responses to concurrent elevated CO_2_ and water deficit remain largely unknown. Specifically, it is unclear whether elevated CO_2_ can mitigate these drought-induced limitations or if drought stress completely overrides CO_2_-induced photosynthetic benefits. Under the combined background of rising atmospheric CO_2_ and intensifying seasonal drought, clarifying the physiological response boundary and drought-adaptation mechanisms of Chinese fir is therefore of considerable ecological and silvicultural significance [23,28].

Here, we conducted a two-factor experiment by exposing Chinese fir saplings to two atmospheric CO_2_ concentrations (400 and 800 ppm) and two soil water regimes (70% field capacity, well-watered; 40% field capacity, well-watered and drought-stressed conditions). We monitored photosynthetic gas exchange over time and quantified chlorophyll fluorescence, photosynthetic pigments, oxidative damage indicators, osmotic adjustment, and antioxidant enzyme activities to comprehensively explore the underlying physiological mechanisms. We hypothesized that: (1) under well-watered conditions, elevated CO_2_ would significantly increase the net photosynthetic rate (*A*_n_) while maintaining photochemical efficiency (*F*v/*F*m, Y(II)) and pigment pools; and (2) under drought stress, the photosynthetic benefits of elevated CO_2_ would be largely negated by stomatal closure, accompanied by specific non-stomatal impairments, namely marked pigment degradation, fluorescence decline, and increased oxidative stress markers (e.g., MDA, proline).

## 2. Results

### 2.1. Effects of Field Capacity and CO_2_ Concentration on Net Photosynthetic Rate (A_n_), Stomatal Conductance (g_s_) and Transpiration Rate (E)

**Figure 1:**
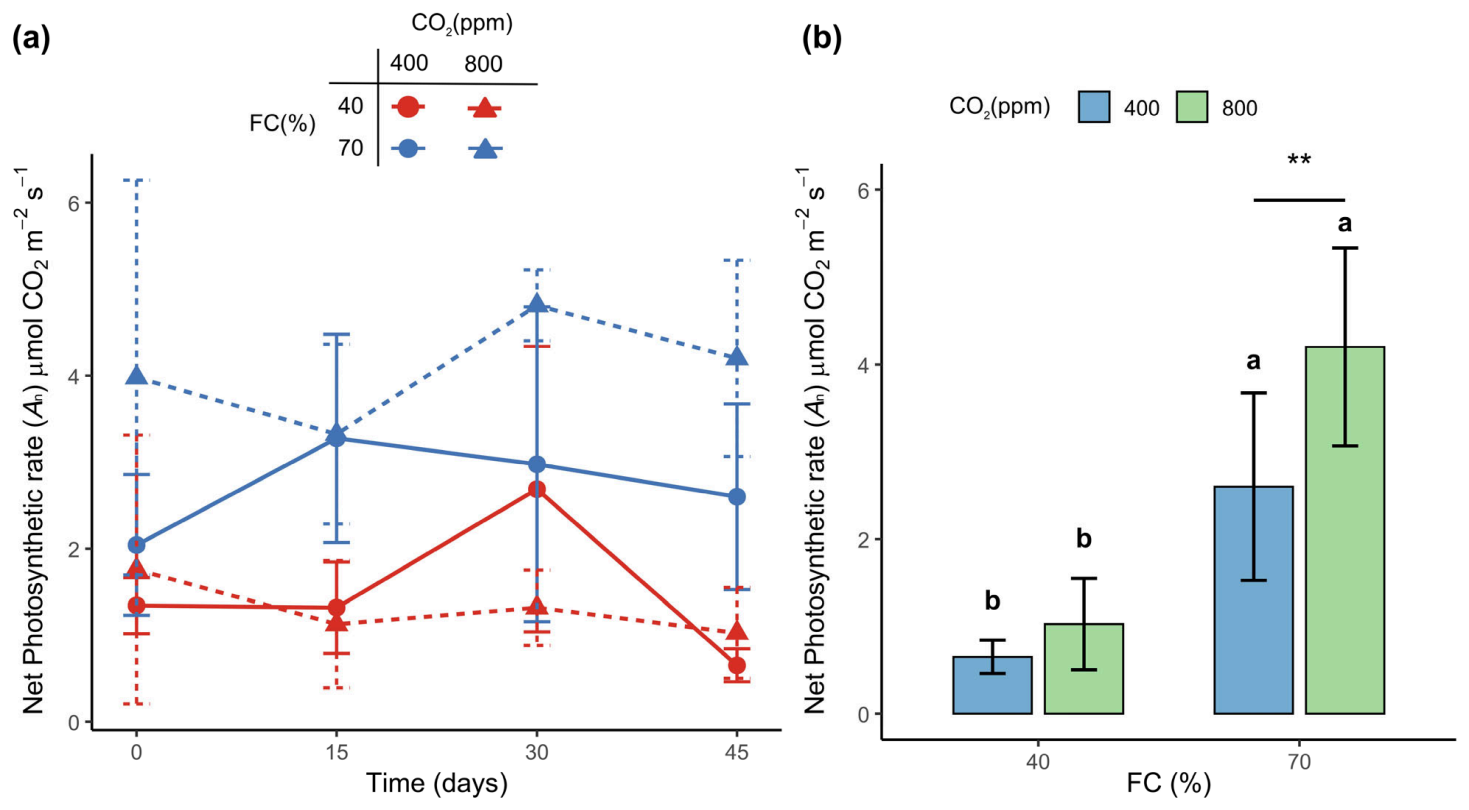
Effects of field capacity (FC) and CO_2_ concentration (CO_2_) on the net photosynthetic rate (*A*_n_) of Chinese fir saplings. (**a**) Temporal changes in *A*_n_ over the 45-day experimental period under different FC (40% and 70%) and CO_2_ treatments (400 and 800 ppm). (**b**) Interactive effects of FC and CO_2_ concentration on *A*_n_ at the final sampling point (Day 45). Values are presented as means ± standard deviation (SD) (n = 4). Different lowercase letters indicate significant differences between soil water regimes (40% vs. 70% FC) within the same CO_2_ level (*p* < 0.05) according to Tukey’s HSD test. Asterisks indicate significant differences between CO_2_ treatments (400 vs. 800 ppm) within the same FC level (*p* < 0.01).

**Figure 2:**
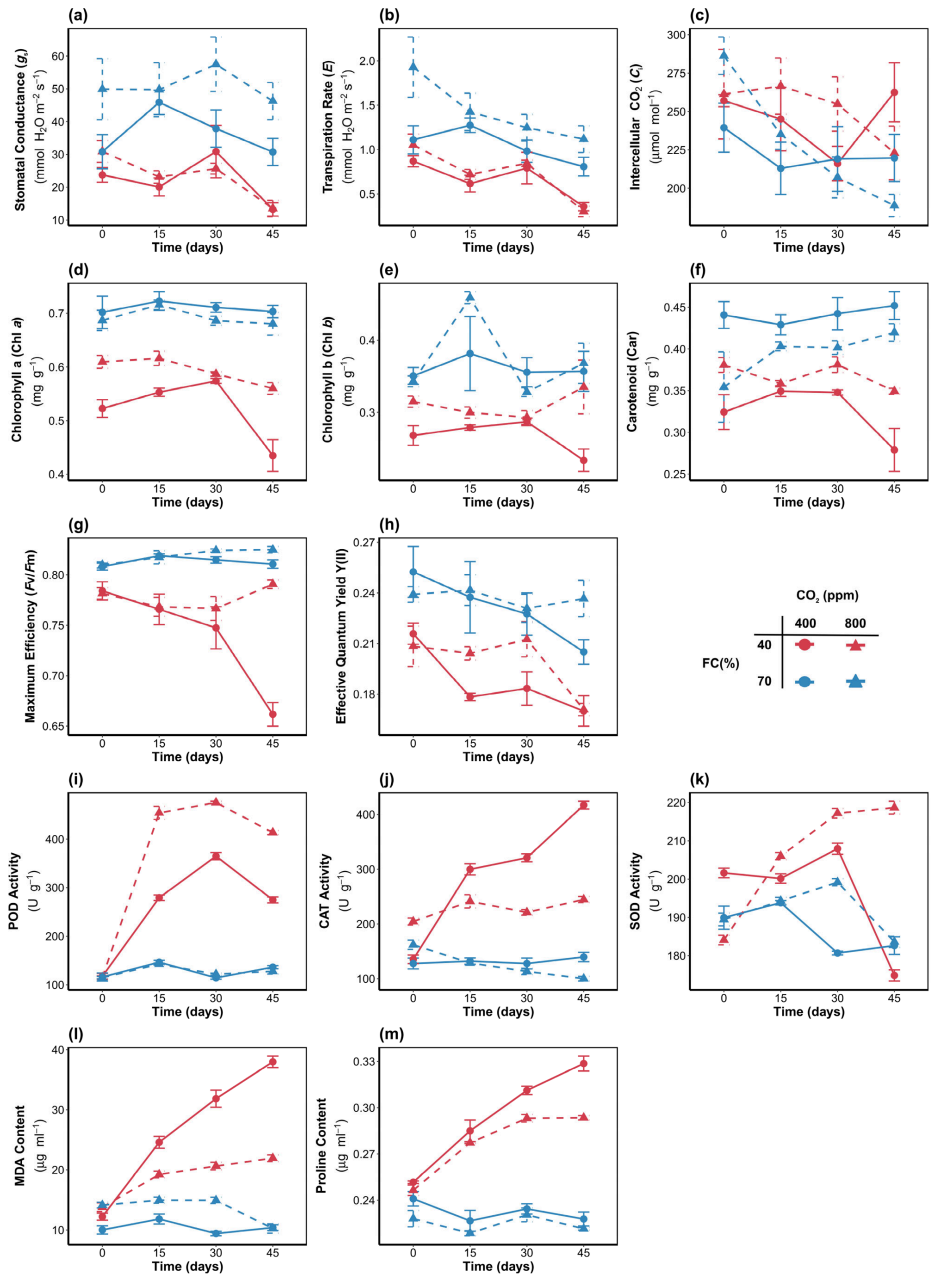
Physiological responses of Chinese fir seedlings to field capacity (FC) and CO_2_ concentration (CO_2_) over time. Temporal changes in (**a**) stomatal conductance (g_s_), (**b**) transpiration rate (*E*), (**c**) intercellular CO_2_ concentration (*C*_i_), (**d**) chlorophyll a content (Chl *a*), (**e**) chlorophyll b content (Chl *b*), (**f**) carotenoid content (Car), (**g**) maximum photochemical efficiency of PSII (*F*v/*F*m), (**h**) effective quantum yield of PSII [Y(II)], (**i**) peroxidase activity (POD), (**j**) catalase activity (CAT), (**k**) superoxide dismutase activity (SOD), (**l**) malondialdehyde content (MDA), and (**m**) proline content (Pro) during the 45-day treatment period. Treatments included two FC levels (40% and 70%) and two CO_2_ concentrations (400 and 800 ppm). Values are presented as means ± SD (n = 4).

Overall, drought stress (40% FC) significantly reduced the *A*_n_ of Chinese fir saplings regardless of CO_2_ concentration (*p* < 0.001, Table 1 and Figure 1). However, elevated CO_2_ significantly increased *A*_n_ under well-watered conditions (70% FC) but exerted no detectable effects under drought stress (*p* < 0.05 and *p* < 0.01 for the CO_2_ main effect and the FC × CO_2_ interaction, respectively; Table 1 and Figure 1). Generally, the temporal patterns and mean values of g_s_, *E* and *C*_i_ in Chinese fir saplings exhibited similar trends in response to the individual and interactive effects of the FC and CO_2_ treatments (Table 1 and Figure 2a–c).

### 2.2. Effects of Field Capacity and CO_2_ Concentration on Chlorophyll Fluorescence, Photosynthetic Pigments, Oxidative Damage Indicators, Osmotic Adjustment, and Antioxidant Enzyme Activities

Drought stress (40% FC) was accompanied by clear declines in chlorophyll fluorescence parameters, including *F*v/*F*m and Y(II), as well as reductions in chlorophyll a (Chl *a*), chlorophyll b (Chl *b*), and carotenoid (Car) contents (Figure 2d–h). By contrast, drought stress significantly increased the activities of antioxidant enzymes, including POD, CAT, and SOD, and elevated the contents of MDA and Pro (Figure 2i–m). Furthermore, CO_2_ concentration significantly affected most of these physiological and biochemical traits, with the exceptions of Y(II) and Car (Table 1). Notably, significant interactive effects between field capacity and CO_2_ concentration (FC × CO_2_) were observed for *F*v/*F*m, Chl *a*, Car, and all oxidative stress-related indicators (POD, CAT, SOD, MDA, and Pro) (Table 1).

### 2.3. Correlation Relationships Among Physiological Traits and Variance Partitioning of Photosynthetic Regulation

**Figure 3:**
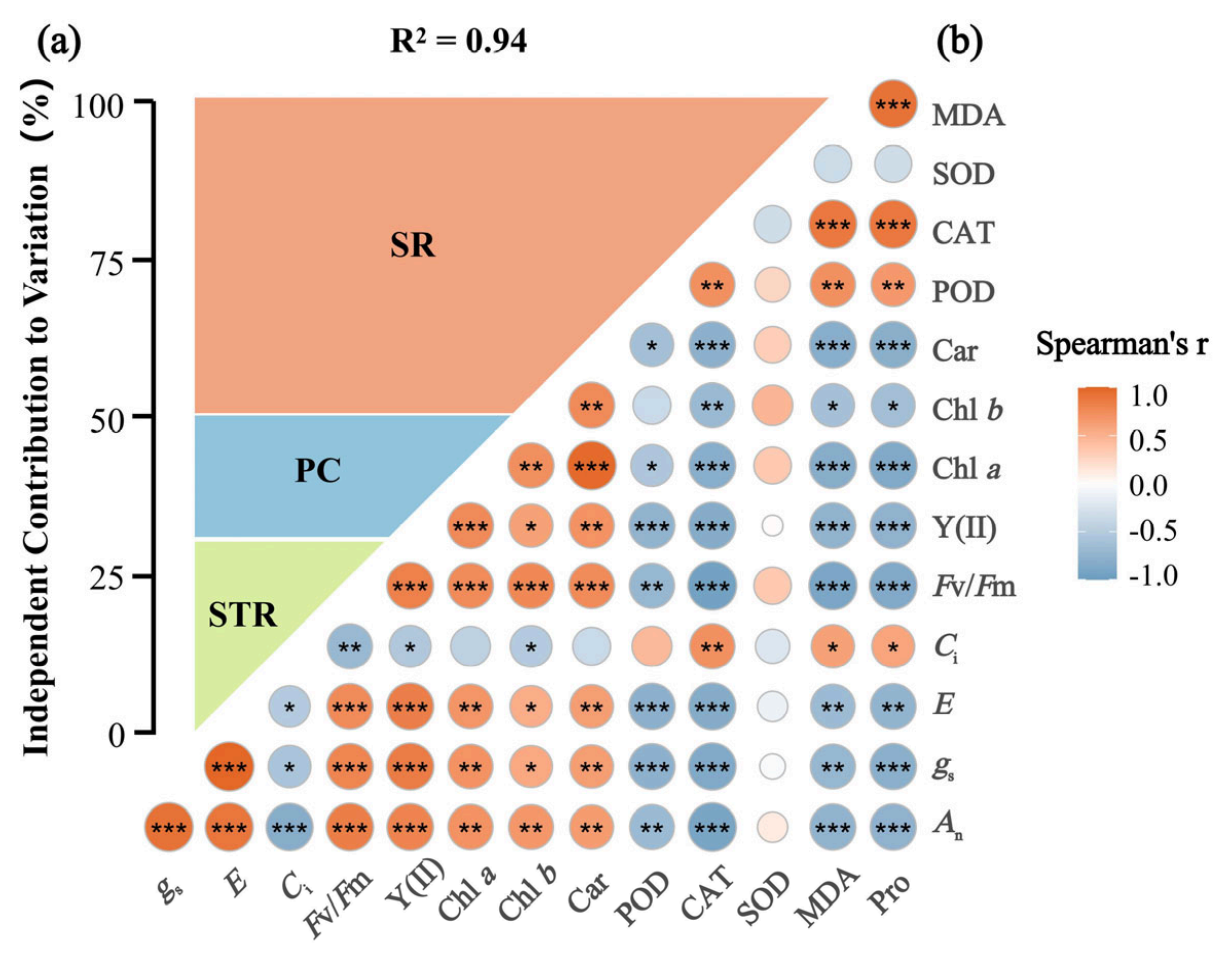
Variance partitioning and Spearman’s rank correlation analysis of physiological traits in Chinese fir seedlings. (**a**) The physiological modules include: SR, stomatal regulation [including stomatal conductance (*g*_s_), transpiration rate (*E*) and intercellular CO_2_ concentration (*C*_i_)]; PC, photosynthetic capacity [including maximum quantum yield of PSII (*F*v/*F*m), effective quantum yield of PSII (Y(II)), chlorophyll a (Chl *a*), chlorophyll b (Chl *b*), and carotenoids (Car)]; and STR, stress response [including superoxide dismutase (SOD), peroxidase (POD), catalase (CAT), malondialdehyde (MDA), and proline (Pro)]. The total explained variance was 94% (R^2^ = 0.94). (**b**) Spearman’s rank correlation matrix among the measured physiological parameters. The color gradient from blue to red indicates correlation coefficients (r) ranging from −1.0 to 1.0. Asterisks indicate significant correlations (* *p* < 0.05; ** *p* < 0.01; *** *p* < 0.001).

Net photosynthetic rate (*A*_n_) was positively correlated with stomatal and photochemical traits, including *g*_s_, *E*, *F*v/*F*m, Y(II), Chl *a*, Chl *b*, and Car, whereas it was negatively correlated with stress-response variables such as SOD, POD, CAT, MDA, and Pro (Figure 3b). Variance partitioning analysis (VPA) further demonstrated that the three predefined functional modules—Stomatal Regulation (SR), Photochemistry (PC), and Stress Response (STR)—jointly explained 94% of the total variation in *A*_n_ (R^2^ = 0.94; Figure 3a). Specifically, SR was the dominant driver, accounting for 49.7% of the independent explained variance, while the non-stomatal modules STR and PC contributed 31.1% and 19.2%, respectively.

### 2.4. Structural Equation Model of Photosynthetic Regulation Under Combined CO_2_ and Drought Treatments

**Figure 4:**
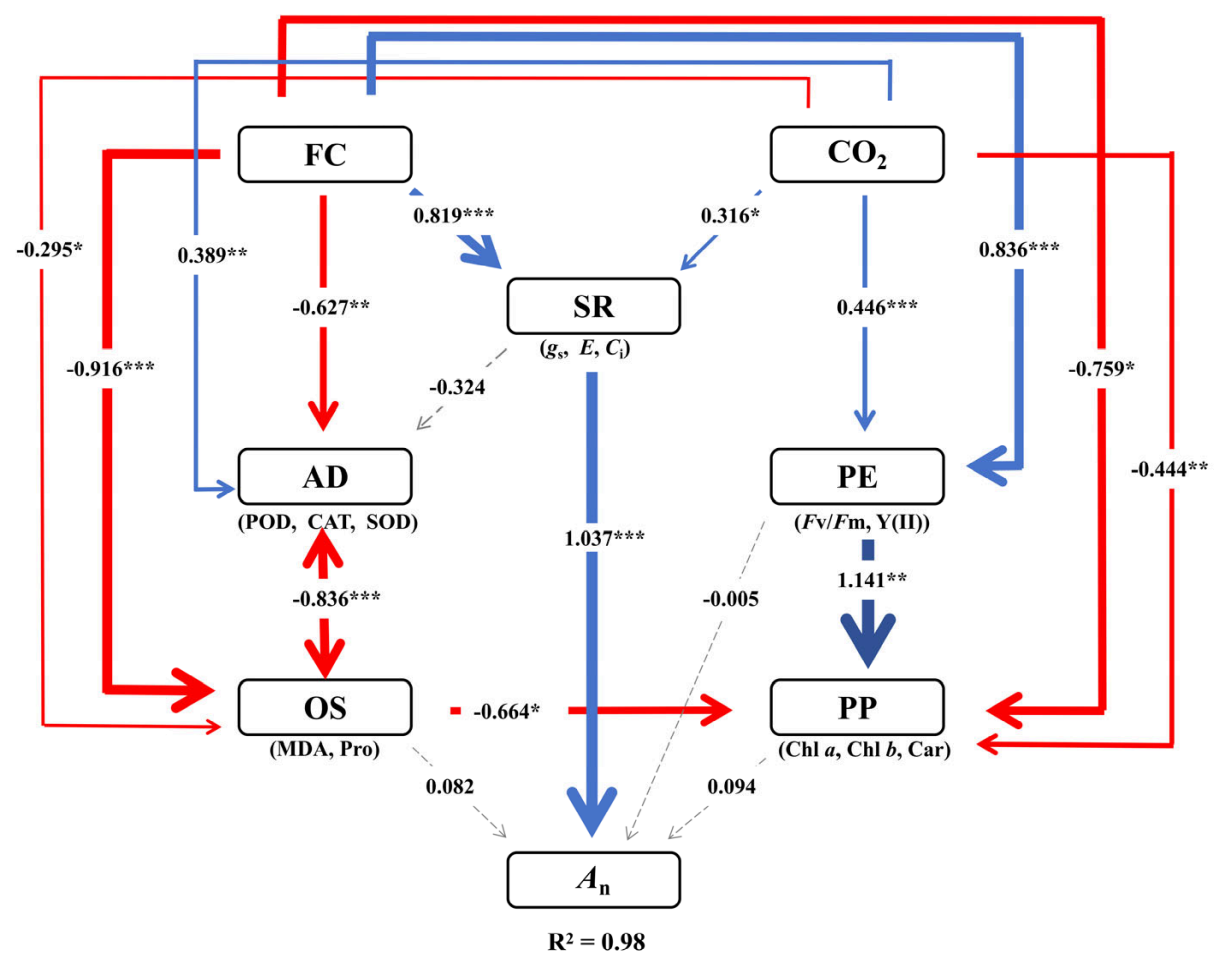
Structural equation model (SEM) describing the pathways by which field capacity (FC) and CO_2_ concentration (CO_2_) regulate the net photosynthetic rate (*A*_n_) of Chinese fir seedlings. The model shows the direct and indirect relationships among environmental factors (FC and CO_2_), stomatal regulation (SR), antioxidant defense (AD), oxidative stress (OS), photochemical efficiency (PE), photosynthetic pigments (PP), and net photosynthetic rate (*A*_n_). Blue and red arrows indicate significant positive and negative effects, respectively, whereas dashed gray arrows indicate non-significant pathways. The width of solid arrows is proportional to the standardized path coefficients. Numbers adjacent to arrows represent standardized path coefficients, and asterisks indicate statistical significance (* *p* < 0.05; ** *p* < 0.01; *** *p* < 0.001). The model explains 98% of the total variance in An (R^2^ = 0.98). The model provided an adequate fit to the data (Fisher’s C = 24.44, *p* = 0.141; Chi-Squared = 25.49, AIC = 96.66). Abbreviations: *g*_s_, stomatal conductance; *E*, transpiration rate; *C*_i_, intercellular CO_2_ concentration; POD, peroxidase; CAT, catalase; SOD, superoxide dismutase; MDA, malondialdehyde; Pro, proline; *F*v/*F*m, maximum quantum yield of PSII; Y(II), effective quantum yield of PSII; Chl *a*, chlorophyll a; Chl *b*, chlorophyll b; Car, carotenoids.

The piecewise structural equation model (pSEM) provided an excellent fit to the data (Fisher’s C = 24.44, *p* = 0.141; Chi-Squared = 25.49, AIC = 96.66) and accurately captured the multivariable causal pathways regulating *A*_n_ (Figure 4). The model demonstrated that field capacity (FC) and CO_2_ concentration (CO_2_) influenced *A*_n_ predominantly through stomatal regulation, which exerted the strongest direct positive effect on final carbon assimilation (standardized path coefficient = 1.037, *p* < 0.001).

Crucially, the pSEM explicitly disentangled the complex interconnected dynamics of non-stomatal impairments under water deficit. Lower FC directly provoked severe oxidative stress (OS) (*p* < 0.001) and suppressed photochemical efficiency (PE) (*p* < 0.001, Figure 4). In turn, this suppressed PE acted as the primary downstream driver undermining the homeostasis of photosynthetic pigments (PP) (*p* < 0.01), while elevated OS exerted a marginally significant negative trend on PP (*p* = 0.072). Furthermore, the model captured a highly significant negative correlated error between OS and antioxidant defense (AD) (γ = −0.836, *p* < 0.001), reflecting their tightly coupled physiological feedback under stress. Although elevated CO_2_ showed significant positive effects on SR (*p* < 0.05), AD (*p* < 0.01), and PE (*p* < 0.001), these partial biochemical mitigations were ultimately overwhelmed by the dominant restrictions imposed by drought-induced stomatal closure and concurrent downstream non-stomatal damage.

## 3. Discussion

### 3.1. Elevated CO_2_ Enhanced Photosynthesis Under Adequate Water Supply

The present study showed that elevated CO_2_ significantly enhanced the net photosynthetic rate (*A*_n_) of Chinese fir saplings under well-watered conditions, supporting the first hypothesis. This result indicates that Chinese fir saplings can effectively convert increased atmospheric CO_2_ availability into enhanced carbon assimilation when soil moisture is sufficient [29]. Under 70% FC, stomatal conductance (*g*_s_) remained at relatively high levels. As elevated CO_2_ increases the external concentration and diffusive gradient, the maintenance of adequate *g*_s_ facilitated CO_2_ diffusion into the leaf, thereby significantly increasing the substrate available for carbon fixation [5,30].

Furthermore, this response suggests that the photosynthetic system of Chinese fir saplings remained functionally intact under adequate water supply. In other words, neither stomatal restriction nor marked biochemical impairment appeared to dominate photosynthetic regulation [15]. Instead, the system retained enough flexibility to respond positively to enhanced carbon availability. Thus, the present results do not suggest that Chinese fir is insensitive to CO_2_ enrichment; rather, they show that the positive effect of elevated CO_2_ can be clearly expressed when water availability does not impose a strong physiological constraint [13].

Furthermore, our chlorophyll fluorescence and pigment data did not indicate marked impairment of the light-harvesting apparatus under well-watered conditions. While we cannot fully discount potential underlying biochemical limitations without *A*/*C*_i_ curve parameters (e.g., maximum rate of Rubisco carboxylation, *V*_cmax_, and maximum electron transport rate, *J*_max_), the maintained *F*v/*F*m and pigment stability suggest that the photochemical system retained enough flexibility to respond positively to enhanced carbon availability. Thus, the present results show that the positive effect of elevated CO_2_ can be clearly expressed when water availability does not impose a strong physiological constraint [13].

The positive response observed under 70% FC was consistent across the 45-day experimental period. However, because our study evaluated saplings over a relatively short timeframe, we cannot rule out the possibility of long-term photosynthetic down-regulation (acclimation) [31]. Nonetheless, for a fast-growing plantation species such as Chinese fir, these short-term results imply that elevated CO_2_ may support rapid carbon gain provided that leaf water relations and photochemical performance remain stable. These results reinforce the concept that the CO_2_ fertilization effect in this species is highly conditional [14,32].

### 3.2. Severe Water Deficit Suppressed Photosynthesis Through Coordinated Stomatal and Photochemical Limitations

In contrast, drought stress caused a marked decline in photosynthetic performance. As explicitly captured by our structural equation model (pSEM), this inhibition cannot be attributed to a single limiting factor. Rather, it reflects a tightly coupled response involving dominant stomatal restriction accompanied by specific non-stomatal constraints, namely photochemical impairment and oxidative damage [15].

At the stomatal level, the pronounced reduction in *g*_s_ and *E* indicates that saplings restricted stomatal opening to limit water loss and maintain short-term hydraulic safety under soil water deficit [12,25]. Our pSEM results robustly confirmed this, showing that gas exchange exerted the strongest direct positive effect on *A*_n_ (standardized coefficient = 1.037). While this stomatal closure acts as a crucial protective mechanism, it simultaneously limits the diffusion of atmospheric CO_2_ into the leaf, severely constraining the substrate supply for carbon fixation [9]. Therefore, a major proportion of the decline in *A*_n_ under drought stress is a direct consequence of this physical restriction on carbon entry.

Concurrently, drought triggered substantial non-stomatal limitations. It is important to precisely distinguish among the various components of non-stomatal impairment, which generally encompass mesophyll, photochemical, and biochemical limitations. Although the absence of *A*/*C*_i_ curves in our study limits our ability to explicitly partition mesophyll conductance and biochemical limitations (e.g., Rubisco carboxylation efficiency) [15], our data provide clear mechanistic evidence for severe photochemical limitation. The pSEM demonstrated that drought directly suppressed maximum photochemical efficiency (*F*v/*F*m) and operational PSII yield (Y(II)). Crucially, this suppressed photochemical performance acted as the primary downstream driver undermining the stability of photosynthetic pigments [19,33]. Meanwhile, elevated MDA and Pro levels, alongside highly significant correlated feedback with antioxidant enzymes (SOD, POD, CAT), underscored severe cellular oxidative stress [16,17]. Taken together, the reduction in *A*_n_ is best understood as a coupled process: stomatal closure physically restricted CO_2_ entry, while parallel oxidative damage and photochemical degradation fundamentally weakened the internal capacity of the leaf to utilize absorbed light [15,33].

### 3.3. Severe Water Deficit Weakened the Photosynthetic Benefit of Elevated CO_2_

A key finding of this study is that the positive effect of elevated CO_2_ on net photosynthesis (*A*_n_) was evident under adequate water supply (70% FC) but did not significantly increase *A*_n_ under drought stress (40% FC) (Figure 1). This highlights that the photosynthetic benefit of elevated CO_2_ in Chinese fir saplings is strongly dependent on soil water status and drought intensity [13,14]. Importantly, while our data robustly capture this physiological response, this inference regarding the FC × CO_2_ interaction operates within the constraints of our growth chamber experimental design. Assuming minimal chamber-specific confounding effects, this pattern clearly demonstrates the conditional nature of the CO_2_ fertilization effect.

This pattern suggests that elevated CO_2_ can enhance photosynthesis provided that the major physiological components supporting carbon assimilation remain functionally intact. First, the positive effect of elevated atmospheric CO_2_ is primarily driven by an increased external concentration and a steeper diffusive gradient. However, the realization of this benefit is regulated by stomatal conductance. Under drought stress, strong stomatal closure restricts this diffusion, drastically reducing internal CO_2_ availability [15]. As a result, the stimulation of photosynthesis by elevated CO_2_ is fundamentally limited when stomatal diffusion is strongly restricted [9]. Second, and perhaps more importantly, the internal photosynthetic capacity also appears to have been compromised under drought stress. The observed increases in oxidative stress indicators, together with pigment loss and reduced PSII efficiency, suggest that the photochemical components of photosynthesis were operating under substantial strain [18,34]. These results suggest that the positive effect of elevated CO_2_ may be limited not only by carbon diffusion, but also by the reduced capacity of the photosynthetic system to transform absorbed energy into stable carbon gain [33,35].

Collectively, these results suggest that drought stress shifted the dominant control over photosynthesis. Under well-watered conditions, carbon availability helped determine photosynthetic performance, so elevated CO_2_ could produce a measurable gain. Under drought stress, however, physiological impairment became a stronger constraint, and the system no longer responded clearly to increased atmospheric CO_2_ [11,28]. These results indicate that elevated CO_2_ retained the potential to enhance photosynthesis, but this effect could be expressed only when water limitation did not impose severe physiological constraints. The fertilization benefit was effectively neutralized once drought stress intensified to the point that both gas exchange and internal photochemical functioning were strongly disrupted [14,32].

### 3.4. Integrated Physiological Mechanisms of Photosynthetic Regulation

To ensure the independence of observations and avoid confounding temporal effects, our multivariate analyses (correlation, variance partitioning, and structural equation modeling) focused on the final sampling point (Day 45). Collectively, these analyses suggest that photosynthetic performance in Chinese fir was regulated through a network of interacting physiological modules, rather than isolated pathways [36].

Specifically, the correlation results showed that net photosynthetic rate (*A*_n_) was positively associated with stomatal and photochemical traits and negatively associated with cellular damage and antioxidant-related indicators. This overall pattern suggests that higher carbon assimilation was closely linked to the coordinated maintenance of stomatal openness, pigment status, and light-use efficiency, whereas lower carbon assimilation occurred alongside greater physiological stress. These results indicate that the drought response should not be interpreted as a change in a single isolated variable. Rather, multiple parts of the photosynthetic system shifted synchronously as drought intensified [12,34].

The variance partitioning results strengthen this interpretation. Stomatal regulation, photochemical capacity, and stress response jointly explained a substantial proportion of the total variation in *A*_n_. This indicates that photosynthesis under the combined CO_2_ and drought treatments was strongly associated with the interaction of several functional domains. This provides a more comprehensive perspective than attributing drought effects solely to stomatal closure or isolated oxidative damage, suggesting instead that the final photosynthetic response emerged from the physiological balance among water loss regulation, internal metabolic stability, and photochemical integrity [35,37].

The pSEM provides an explicit statistical framework for these complex associations. The model indicates that field capacity (FC) influenced *A*_n_ predominantly through pathways involving stomatal regulation and downstream photochemical processes, while the role of elevated CO_2_ under drought stress was comparatively limited. Particularly important is the absence of a significant pathway indicating that elevated CO_2_ could offset oxidative stress or preserve pigment homeostasis under severe water deficit. In this framework, the influence of elevated CO_2_ became secondary under drought stress, as the physiological modules most strongly associated with *A*_n_ shifted from a relatively substrate-responsive state to a stress-dominated state [38].

### 3.5. Implications for Chinese Fir Performance Under Future Climate Conditions and Study Limitations

The present findings provide mechanistic insights into how Chinese fir saplings may respond to future climate scenarios characterized by rising atmospheric CO_2_ and more frequent water deficit events [1,26]. While elevated CO_2_ is often expected to stimulate plant carbon gain [5,39], our results emphasize that this expectation must be treated as highly conditional [32]. In Chinese fir, the positive effect of elevated CO_2_ was apparent only when water availability remained adequate. Once water deficit intensified to a severe level, the net photosynthetic benefit was substantially neutralized.

This finding is particularly relevant for Chinese fir because the species is widely planted and highly productive under suitable conditions, yet physiologically sensitive to water limitation [22,23]. As a shallow-rooted plantation conifer, its performance under climate change is likely to depend not only on rising atmospheric CO_2_, but also on whether soil water deficit stays within a range that the photochemical system can tolerate without major internal damage [24,25]. Accordingly, predicting future plantation productivity on the basis of CO_2_ enrichment alone may overestimate actual carbon gains [11].

Crucially, these implications must be interpreted with caution due to several methodological limitations. First, this study was conducted on potted saplings in climate-controlled chambers over a relatively short period (45 days) under fixed light conditions (PPFD of 500 μmol m^−2^ s^−1^) and a simplified two-factor design. Thus, these results represent short-term physiological responses rather than long-term ecological acclimation in mature forest stands. Second, the absence of detailed *A*/*C*_i_ curve parameters limits our ability to explicitly quantify underlying biochemical restrictions (e.g., *V*_cmax_ and *J*_max_). Finally, while our data capture robust biological trends, the allocation of treatments within growth chambers necessitates that inferences regarding the CO_2_ main effect and interactive effects be drawn with the assumption of minimal chamber-specific confounding factors.

From a management perspective, the results imply that the potential benefit of elevated CO_2_ should not be viewed as an automatic buffer against drought stress [11,23]. If severe seasonal water deficits become more frequent, maintaining plantation productivity may depend more on improving resilience to water limitation than relying on the passive fertilization effect of atmospheric CO_2_ [12]. Future assessments of Chinese fir productivity should account for the possibility that drought stress can fundamentally weaken the expression of CO_2_-induced photosynthetic gains. This conclusion is not a broad claim that CO_2_ enrichment is globally unimportant, but rather a species- and stress-specific inference supported by the experimental evidence [32].

## 4. Materials and Methods

### 4.1. Plant Materials

One-year-old healthy saplings of Chinese fir (*Cunninghamia lanceolata*) were obtained from a third-generation clonal seed orchard located in Fujian Province, China, to ensure a consistent genetic background. Saplings were grown individually in plastic pots (35 cm in diameter and 35 cm in height). To provide adequate aeration and nutrient retention, the pots were filled with a 1:1 (*v*/*v*) mixture of native yellow soil and commercial nursery substrate (primarily humus soil). The final growth medium contained 0.51 g kg^−1^ total nitrogen and 0.18 g kg^−1^ total phosphorus. Prior to treatment initiation, all saplings were cultivated under natural light conditions and irrigated regularly to field capacity to ensure uniform growth status.

Based on strict morphological uniformity criteria, a total of 50 saplings were selected (mean height: 61.78 ± 4.60 cm; mean basal diameter: 9.17 ± 1.24 mm). To avoid the confounding effects of wounding stress from destructive sampling on the time-course gas exchange measurements, we implemented a spatially separated sampling strategy. Specifically, 48 saplings were randomly assigned to the four treatment combinations (n = 12 saplings per treatment), leaving 2 saplings as reserves. Within each treatment, four designated saplings (n = 4) were exclusively reserved for repeated, non-destructive measurements (gas exchange and chlorophyll fluorescence) on fixed, intact needles throughout the 45-day period. The remaining eight saplings per treatment served as an independent pool for destructive biochemical sampling (pigments, antioxidants, and osmolytes) across the four time points, thereby ensuring the absolute independence and accuracy of the physiological monitoring.

### 4.2. Experimental Design

The experiment was conducted in four identical climate-controlled CO_2_ growth chambers (CIC250, Fujian Jiupo Biotechnology Co., Ltd., Fuzhou, China) using a two-factor completely randomized design. Two atmospheric CO_2_ concentrations were established: ambient CO_2_ (400 ppm) and elevated CO_2_ (800 ppm). Two soil water regimes were imposed based on field capacity (FC): well-watered conditions (70% FC) and drought stress (40% FC). Accordingly, four treatment combinations were generated: 400 ppm CO_2_ + 70% FC, 400 ppm CO_2_ + 40% FC, 800 ppm CO_2_ + 70% FC, and 800 ppm CO_2_ + 40% FC. Previous studies have robustly demonstrated that substantial soil moisture limitation induces significant physiological stress, photosynthetic inhibition, and structural adjustments in *C. lanceolata* saplings [20,25,27]. Therefore, the 40% FC treatment in our study was specifically implemented to represent a severe water deficit scenario.

Crucially, to explicitly avoid pseudoreplication caused by potential “chamber effects”, we implemented a strict calibration and spatial rotation strategy. Prior to the experiment, the photosynthetically active radiation (PAR), temperature, and humidity across all chambers were independently cross-calibrated. During the 45-day treatment period, the entire set of saplings, along with their corresponding CO_2_ concentration programs, were systematically rotated between the four chambers every 15 days. Furthermore, the positions of the pots within each chamber were randomly rotated weekly to eliminate internal edge effects.

The environmental conditions in the chambers were maintained at 25/22 °C (day/night), 60% relative humidity, a 16 h photoperiod, and a photosynthetic photon flux density (PPFD) of 500 µmol m^−2^ s^−1^. This specific PPFD level was selected as it is sufficient to drive active photosynthesis for shade-tolerant Chinese fir saplings while preventing high-light-induced photoinhibition in confined chamber environments.

To determine 100% FC, the soil mixture in representative pots was fully saturated with water and allowed to drain freely by gravity for 48 h until the weight became constant. The target mass for each pot at 70% and 40% FC was then calculated based on this saturated water weight, dry soil weight, and empty pot weight. During the experiment, soil water content was monitored daily using a portable soil moisture meter (TZS-2X, Zhejiang Top Cloud-agri Technology Co., Ltd., Hangzhou, China) and gravimetric weighing. Pots were weighed daily at 18:00 and rewatered as needed to maintain the target FC. To minimize soil evaporation and ensure that water loss was primarily due to plant transpiration, the soil surface of each pot was covered with a layer of dry quartz sand. As described in the plant materials section, physiological measurements and destructive sampling were conducted at 0, 15, 30, and 45 d after the initiation of the treatments using the spatially separated saplings.

### 4.3. Determination of Physiological Traits

#### 4.3.1. Gas Exchange and Chlorophyll Fluorescence

Leaf gas-exchange parameters were measured on intact, fully expanded needles using a CIRAS-3 portable photosynthesis system (PP Systems, Amesbury, MA, USA) between 08:00 and 11:30 h. Following best practice guidelines for gas-exchange measurements [40], the leaf chamber conditions were strictly controlled: reference CO_2_ concentrations were set to match the respective growth treatments (400 or 800 ppm), PPFD was maintained at 500 µmol m^−2^ s^−1^, leaf temperature was set to 25 °C, air flow rate at 300 mL min^−1^, and vapor pressure deficit (VPD) was maintained at approximately 1.0–1.2 kPa. Net photosynthetic rate (*A*_n_), stomatal conductance (*g*_s_), intercellular CO_2_ concentration (*C*_i_) and transpiration rate (*E*) were recorded only after parameters stabilized (typically 3–5 min). Because *Cunninghamia lanceolata* is a conifer, the projected leaf area of the specific needles enclosed in the cuvette was determined immediately post-measurement using a scanner and ImageJ software (JS version, NIH, Bethesda, MD, USA). The initial gas-exchange data were then recalculated based on this precise projected leaf area.

Chlorophyll fluorescence was measured on the same leaves using a Yaxin-1165 fluorometer (Yaxin, Beijing, China). After 30 min of dark adaptation, the maximum photochemical efficiency of PSII (*F*v/*F*m) was determined using a saturating pulse. The effective quantum yield of PSII [Y(II)] was measured under actinic light equivalent to the growth conditions.

#### 4.3.2. Photosynthetic Pigments

Fresh leaf samples (approx. 0.1 g) were extracted with 10 mL of an acetone–ethanol solution (1:1, *v*/*v*) in the dark at room temperature until the tissues turned completely white (approximately 24 h). Absorbance was measured at 663, 645, and 470 nm using a SpectraMax iD5 microplate reader (Molecular Devices, San Jose, CA, USA). The concentrations of chlorophyll a (Chl *a*), chlorophyll b (Chl *b*), and carotenoids (Car) were calculated using standard equations [41] and expressed as mg g^−1^ fresh weight (FW).

#### 4.3.3. Oxidative Stress Indicators and Antioxidant Enzyme Activities

To assess oxidative stress and antioxidant responses, fresh leaf tissues (approx. 0.2 g) were homogenized in 2 mL of 0.05 mol L^−1^ phosphate buffer (pH 7.8) in an ice bath. The homogenate was centrifuged at 10,000× *g* for 20 min at 4 °C, and the resulting supernatant was used for subsequent assays. Malondialdehyde (MDA) content was determined using the thiobarbituric acid (TBA) method to estimate the degree of membrane lipid peroxidation and expressed as nmol g^−1^ FW. Proline (Pro) content was quantified using an acid-ninhydrin colorimetric assay kit (Solarbio, Beijing, China) and expressed as µg g^−1^ FW.

The activities of key antioxidant enzymes were determined using commercial assay kits (Solarbio, Beijing, China) in accordance with the manufacturer’s protocols. Superoxide dismutase (SOD) activity was measured using the nitroblue tetrazolium (NBT) photochemical reduction method, peroxidase (POD) activity by the guaiacol colorimetric method, and catalase (CAT) activity by the ultraviolet absorption method. All enzymatic activities were normalized to fresh leaf mass and expressed as U g^−1^ FW.

### 4.4. Statistical Analysis

All statistical analyses were performed in R version 4.4.2. Data are presented as mean ± standard deviation (SD). To strictly align with our spatially separated sampling strategy, distinct analysis of variance (ANOVA) approaches were applied. For non-destructive traits measured repeatedly on the same intact saplings over time (gas exchange and chlorophyll fluorescence), a repeated-measures ANOVA was used, with CO_2_ concentration, field capacity (FC), and sampling time as fixed effects, and the individual sapling as the repeated unit. Conversely, for biochemical traits obtained through destructive sampling of independent saplings at each time point (pigments, oxidative indicators, and enzymes), a standard three-way ANOVA was conducted. When necessary, post hoc multiple comparisons (e.g., Tukey’s HSD) were performed to assess differences among treatment combinations at each sampling time. Differences were considered statistically significant at *p* < 0.05, *p* < 0.01, and *p* < 0.001.

To evaluate the multivariate relationships, Spearman’s rank correlation coefficients were calculated for the Day 45 dataset and visualized as a lower-triangular matrix using the ggcorrplot package, with significance levels (*p* < 0.05, *p* < 0.01, *p* < 0.001) indicated by asterisks. Crucially, to strictly ensure the independence of observations and avoid pseudoreplication caused by pooling time-series data, advanced multi-variable modeling was performed using exclusively the cross-sectional data collected at the final sampling point (Day 45). Prior to modeling, variables were normalized using min–max scaling to ensure comparability. Variance Partitioning Analysis (VPA) was conducted using the glmm.hp package based on an ordinary multiple linear regression model (lm). This allowed us to quantify the independent explained variance (I.perc) of three predefined functional modules on net photosynthetic rate (*A*_n_): Stomatal Regulation (*g*s, *E*, *C*_i_), Photochemistry (Chl *a*, Chl *b*, Car, *F*v/*F*m, Y(II)), and Stress Response (POD, CAT, SOD, MDA, Pro).

Furthermore, piecewise Structural Equation Modeling (pSEM) was constructed using the piecewiseSEM package to disentangle the direct and indirect causal pathways linking environmental drivers (FC and CO_2_) to physiological regulation and final photosynthetic performance. Crucially, to prevent data leakage and avoid statistical circularity in model fitting, composite variables representing distinct physiological subsystems (SR, AD, PE, OS and PP) were constructed by extracting the first principal component (PC1) via Principal Component Analysis (PCA) on their respective constituent indicators. To ensure consistent biological directionality, the sign of PC1 was automatically evaluated against a primary reference indicator for each module (e.g., *g*_s_ for SR, MDA for Stress) and inverted if negatively correlated. These standardized composite variables were subsequently used to evaluate individual causal paths within the pSEM framework using standard linear models, reflecting the independent nature of the Day 45 dataset.

## Figures and Tables

**Table 1:** Analysis of variance (ANOVA) for the effects of time, field capacity (FC), CO_2_ concentration (CO_2_), and their interactions on the physiological traits of Chinese fir seedlings. To account for the experimental design, non-destructive traits (gas exchange and chlorophyll fluorescence) measured on the same leaves were analyzed using Repeated-Measures ANOVA, whereas destructively sampled biochemical traits (pigments, antioxidants, osmolytes, and MDA) from different replicates at each time point were analyzed using standard Three-way ANOVA. Values presented are F-values. Significance levels are indicated by asterisks: * *p* < 0.05, ** *p* < 0.01, *** *p* < 0.001.

Traits	Time	FC	CO_2_	Time × FC	Time × CO_2_	FC × CO_2_	Time × FC × CO_2_
Gas exchange & Fluorescence (Repeated-measures)							
*A*_n_	1.69	48.95 ***	4.15 *	0.64	1.09	7.35 **	1.17
*g*_s_	3.71 **	62.45 ***	8.92 **	1.30	0.56	6.20 *	0.87
*E*	11.48 ***	56.50 ***	10.05 **	1.41	1.59	4.75	0.75
*C*_i_	4.47 **	7.12 *	0.58	1.23	2.86 *	0.01	1.33
*F*v/*F*m	5.67 **	168.74 ***	23.16 ***	9.25 ***	14.65 ***	12.28 ***	9.91 ***
Y(II)	7.14 ***	63.90 ***	3.28	0.92	1.65	0.30	1.58
Biochemical traits (Three-way ANOVA)							
Chl *a*	9.55 ***	326.9 ***	11.76 ***	4.66 **	2.31	31.58 ***	2.18
Chl *b*	2.96 ***	57.58 ***	7.52 **	3.05 *	2.17	2.12	2.26
Car	1.13	74.75 ***	0.06	4.27 **	0.80	28.61 ***	1.90
POD	602.7 ***	4140 ***	328.3 ***	504.2 ***	42.87 ***	348.5 ***	46.68 ***
CAT	53.35 ***	1173 ***	85.88 ***	102.1 ***	73.60 ***	60.14 ***	21.06 ***
SOD	54.35 ***	262.2 ***	103.4 ***	30.22 ***	82.51 ***	13.41 ***	78.98 ***
MDA	84.52 ***	857.34 ***	38.94 ***	117.45 ***	37.85 ***	219.54 ***	20.62 ***
Pro	41.02 ***	838.4 ***	37.77 ***	60.15 ***	2.19	4.88 *	4.17 *

Note: *A*_n_, net photosynthetic rate; *g*_s_, stomatal conductance; *E*, transpiration rate; *C*_i_, intercellular CO_2_ concentration; *F*v/*F*m, maximum quantum yield of PSII; Y(II), effective quantum yield of PSII; Chl *a*, chlorophyll a; Chl *b*, chlorophyll b; Car, carotenoids; POD, peroxidase; CAT, catalase; SOD, superoxide dismutase; MDA, malondialdehyde; Pro, proline.

## Data Availability

The original contributions presented in this study are included in the article. Further inquiries can be directed to the corresponding authors.

## References

[1] IPCC (2023). Climate Change 2021—The Physical Science Basis: Working Group I Contribution to the Sixth Assessment Report of the Intergovernmental Panel on Climate Change.

[2] Meinshausen M., Nicholls Z.R.J., Lewis J., Gidden M.J., Vogel E., Freund M., Beyerle U., Gessner C., Nauels A., Bauer N. (2020). The shared socio-economic pathway (SSP) greenhouse gas concentrations and their extensions to 2500. Geosci. Model Dev..

[3] Dai A. (2013). Increasing drought under global warming in observations and models. Nat. Clim. Change.

[4] Huang J., Yu H., Guan X., Wang G., Guo R. (2016). Accelerated dryland expansion under climate change. Nat. Clim. Change.

[5] Long S.P., Ainsworth E.A., Rogers A., Ort D.R. (2004). Rising atmospheric carbon dioxide: Plants FACE the future. Annu. Rev. Plant Biol..

[6] Ainsworth E.A., Rogers A. (2007). The response of photosynthesis and stomatal conductance to rising [CO_2_]: Mechanisms and environmental interactions. Plant Cell Environ..

[7] Zhao M., Running S.W. (2010). Drought-induced reduction in global terrestrial net primary production from 2000 through 2009. Science.

[8] Beer C., Reichstein M., Tomelleri E., Ciais P., Jung M., Carvalhais N., Rödenbeck C., Arain M.A., Baldocchi D., Bonan G.B. (2010). Terrestrial gross carbon dioxide uptake: Global distribution and covariation with climate. Science.

[9] Chaves M.M., Pereira J.S., Marôco J., Rodrigues M.L., Ricardo C.P.P., Osório M.L., Carvalho I., Faria T., Pinheiro C. (2002). How plants cope with water stress in the field? Photosynthesis and growth. Ann. Bot..

[10] Keenan T.F., Hollinger D.Y., Bohrer G., Dragoni D., Munger J.W., Schmid H.P., Richardson A.D. (2013). Increase in forest water-use efficiency as atmospheric carbon dioxide concentrations rise. Nature.

[11] Fatichi S., Leuzinger S., Paschalis A., Langley J.A., Donnellan Barraclough A., Hovenden M.J. (2016). Partitioning direct and indirect effects reveals the response of water-limited ecosystems to elevated CO_2_. Proc. Natl. Acad. Sci. USA.

[12] Brodribb T.J., Powers J., Cochard H., Choat B. (2020). Hanging by a thread? Forests and drought. Science.

[13] Wullschleger S.D., Tschaplinski T.J., Norby R.J. (2002). Plant water relations at elevated CO_2_—Implications for water-limited environments. Plant Cell Environ..

[14] Leakey A.D.B., Ainsworth E.A., Bernacchi C.J., Rogers A., Long S.P., Ort D.R. (2009). Elevated CO_2_ effects on plant carbon, nitrogen, and water relations: Six important lessons from FACE. J. Exp. Bot..

[15] Flexas J., Medrano H. (2002). Drought-inhibition of photosynthesis in C3 plants: Stomatal and non-stomatal limitations revisited. Ann. Bot..

[16] Cruz de Carvalho M.H. (2008). Drought stress and reactive oxygen species: Production, scavenging and signaling. Plant Signal. Behav..

[17] Hasanuzzaman M., Bhuyan M.H.M., Zulfiqar F., Raza A., Mohsin S., Mahmud J., Fujita M., Fotopoulos V. (2020). Reactive oxygen species and antioxidant defense in plants under abiotic stress: Revisiting the crucial role of a universal defense regulator. Antioxidants.

[18] Xu Z., Jiang Y., Zhou G. (2015). Response and adaptation of photosynthesis, respiration, and antioxidant systems to elevated CO_2_ with environmental stress in plants. Front. Plant Sci..

[19] Liu X., Zhang H., Wang J., Wu X., Ma S., Xu Z., Zhou T., Xu N., Tang X., An B. (2019). Increased CO_2_ concentrations increasing water use efficiency and improvement PSII function of mulberry sapling leaves under drought stress. J. Plant Interact..

[20] Gao S., Cai Z.Y., Yang C.C., Luo J.X., Zhang S. (2021). Provenance-specific ecophysiological responses to drought in *Cunninghamia lanceolata*. J. Plant Ecol..

[21] National Forestry and Grassland Administration (2019). China Forest Resources Report (2014–2018).

[22] Xie X., Cui J., Shi W., Liu X., Tao X., Wang Q., Xu X. (2016). Biomass partition and carbon storage of *Cunninghamia lanceolata* chronosequence plantations in Dabie mountains in east China. Dendrobiology.

[23] Fang X., Lin T., Zhang B., Lai Y., Chen X., Xiao Y., Xie Y., Zhu J., Yang Y., Wang J. (2022). Regulating carbon and water balance as a strategy to cope with warming and drought climate in *Cunninghamia lanceolata* in southern China. Front. Plant Sci..

[24] Li L., Deng X., Zhang T., Tian Y., Ma X., Wu P. (2022). Propagation methods decide root architecture of Chinese fir: Evidence from tissue culturing, rooted cutting and seed germination. Plants.

[25] Feng J., Yao Y., He Y., Wang P., Hu H., Zhang S. (2025). Hydraulic strategies of *Cunninghamia lanceolata* under drought are shaped by native drought conditions. For. Res..

[26] Yu M., Li Q., Hayes M.J., Svoboda M.D., Heim R.R. (2014). Are droughts becoming more frequent or severe in China based on the standardized precipitation evapotranspiration index: 1951–2010?. Int. J. Climatol..

[27] Dong T., Duan B., Zhang S., Korpelainen H., Niinemets Ü., Li C. (2016). Growth, biomass allocation and photosynthetic responses are related to intensity of root severance and soil moisture conditions in the plantation tree *Cunninghamia lanceolata*. Tree Physiol..

[28] Duan H., Duursma R.A., Huang G., Smith R.A., Choat B., O’Grady A.P., Tissue D.T. (2014). Elevated [CO_2_] does not ameliorate the negative effects of elevated temperature on drought-induced mortality in Eucalyptus radiata seedlings. Plant Cell Environ..

[29] Ainsworth E.A., Long S.P. (2005). What have we learned from 15 years of free-air CO_2_ enrichment (FACE)? A meta-analytic review of the responses of photosynthesis, canopy properties and plant production to rising CO_2_. New Phytol..

[30] Drake B.G., Gonzalez-Meler M.A., Long S.P. (1997). More efficient plants: A consequence of rising atmospheric CO_2_?. Annu. Rev. Plant Physiol. Plant Mol. Biol..

[31] Medlyn B.E., Barton C.V.M., Broadmeadow M.S.J., Ceulemans R., De Angelis P., Forstreuter M., Freeman M., Jackson S.B., Kellomäki S., Laitat E. (2001). Stomatal conductance of forest species after long-term exposure to elevated CO_2_ concentration: A synthesis. New Phytol..

[32] Körner C. (2006). Plant CO_2_ responses: An issue of definition, time and resource supply. New Phytol..

[33] Lawlor D.W., Tezara W. (2009). Causes of decreased photosynthetic rate and metabolic capacity in water-deficient leaf cells: A critical evaluation of mechanisms and integration of processes. Ann. Bot..

[34] Pinheiro C., Chaves M.M. (2011). Photosynthesis and drought: Can we make metabolic connections from available data?. J. Exp. Bot..

[35] Chaves M.M., Flexas J., Pinheiro C. (2009). Photosynthesis under drought and salt stress: Regulation mechanisms from whole plant to cell. Ann. Bot..

[36] Cramer G.R., Urano K., Delrot S., Pezzotti M., Shinozaki K. (2011). Effects of abiotic stress on plants: A systems biology perspective. BMC Plant Biol..

[37] Osakabe Y., Osakabe K., Shinozaki K., Tran L.S.P. (2014). Response of plants to water stress. Front. Plant Sci..

[38] McDowell N., Pockman W.T., Allen C.D., Breshears D.D., Cobb N., Kolb T., Plaut J., Sperry J., West A., Williams D.G. (2008). Mechanisms of plant survival and mortality during drought: Why do some plants survive while others succumb to drought?. New Phytol..

[39] Walker A.P., De Kauwe M.G., Bastos A., Belmecheri S., Georgiou K., Keeling R.F., McMahon S.M., Medlyn B.E., Moore D.J.P., Norby R.J. (2021). Integrating the evidence for a terrestrial carbon sink caused by increasing atmospheric CO_2_. New Phytol..

[40] Ofori-Amanfo K.K., Klem K., Veselá B., Holub P., Agyei T., Juráň S., Grace J., Marek M.V., Urban O. (2023). The effect of elevated CO_2_ on photosynthesis is modulated by nitrogen supply and reduced water availability in Picea abies. Tree Physiol..

[41] Lichtenthaler H.K. (1987). Chlorophylls and carotenoids: Pigments of photosynthetic biomembranes. Methods Enzymol..

